# Molecular docking analysis of Cianidanol fromGinkgo biloba with HER2+ breast cancer target

**DOI:** 10.6026/97320630014482

**Published:** 2018-11-21

**Authors:** Abiodun Julius Arannilewa1, Oluwaseun Suleiman Alakanse, Adesola Oluwaseun Adesola, Oluwaseyi Israel Malachi, Ifedayo Michael Obaidu, Emmanuel Ekun Oluwafemi, Emmanuel Damilola Afolayan, Patricia Folakemi Afere, Kayode Abdullateef Ayuba, Tolulope Oluwafemi Bolarinwa, George Oche Ambrose

**Affiliations:** 1 Department of Biochemistry, University of Ilorin, Ilorin, Nigeria; 2 Department of Biochemistry, Ekiti State University, Ado Ekiti,Nigeria; 3 Department of Biochemistry, Federal University of Technology, Akure, Nigeria; 4 Department of Biochemistry, AdekunleAjasin University, Akungba Akoko, Nigeria; 5 Food Production Department, Goodfoods Inc., Montreal, QC, Canada; 6 Department ofBiochemistry, University of Ibadan, Ibadan, Nigeria

**Keywords:** Cianidanol, Ginkgo biloba, HER2

## Abstract

HER2 is a known therapeutic target for about 30% of breast cancer patients where HER2 is over expressed and this is referred to as HER2 positive breast cancer. This subtype is characterized by a clinical behavior know to be especially aggressive. Improved HER2 targeting agents such as trastuzumab, pertuzumb, lapatinib and ado-trastuzumab emtansine are available. Some patients have shown no response to treatment while others show progress to these agents. Therefore, it is of interest to screen HER2+ with phyto-chemical lead compound from Ginkgo biloba using molecular docking techniques. We screened 25 phyto-chemicals from literature with HER2+. Results show that cianidanol have an acceptable binding energy of (-8.2kcal/mol). Thus, we report the binding properties of cianidanol with HER2+.

## Background

Human Epidermal Growth Factor Receptor type 2 (HER2) belongs to the family of human epidermal growth factor receptors (HER/EGFR/ERBB), which also includes HER1, HER3, and HER4. They are a special family of oncogenic proteins whose amplification has been shown to play important roles in the development and progression of the certain aggressive type of breast cancer. Recently, HER2 has become an important biomarker and therapeutic target for about 30% of cases of breast cancer in patients. 1 Despite recent breakthroughs, breast cancer remains the most prevalent type of cancer in women and the second most deadly disease in advanced countries 2. HER2 is of particular interest in breast cancer because, in one of its subtype (amounting to about 15 - 20 % of all cases), it is over-expressed, giving it the name HER2+ breast cancer. This subtype is particularly characterized by a clinical behavior known to be especially aggressive 3. In about 50 % of cases where this receptor is over-expressed, estrogen receptor (ER) and/or progesterone receptor (PR) is also over-expressed 4. While considering this type of breast carcinoma, the development of targeted therapies has considerably improved its prognosis. Although advanced cases are still mostly considered incurable and there is a wide variation in survival among patients 5

Hitherto, there are no known activating ligands of HER2 directly implicated in triggering its signaling cascade, but there are reports that the receptor is activated by homo- and heterodimerization with other known HER family receptors. These include the HER3, HER4 and HER1 6,7. Dimerization of HER2 and HER3 has been shown to be the most potent oncogenic pair, leading to activation of their kinases with subsequent activation of downstream signaling pathways which includes the phosphatidylinositol-3-kinase (PI3K)/Akt and Mitogen-Activated Protein Kinase (MAPK) 6-9. Consequently, the activated signaling cascade promotes expression of more oncogenes that are involved in orchestrating the survival of tumor cells, their proliferation and differentiation, thus fostering tumor progression 9,10. Accurately assessing HER2 status is crucial in decision making involving treatment for patients with breast cancer. A false-negative status for HER2 may lead to decisions where anti-HER2-directed therapy is omitted as much as a falsepositive status may bring about unnecessary administration of cost-ineffective and extended treatment with no known benefits 11.

Research involving HER2 targeting agents has progressed over the years with discoveries showing this to be one of the most productive research interests in oncogenic drug development. Trastuzumab targeted therapy as well as agents such as pertuzumab, lapatinib and ado-trastuzumab emtansine (T-DM1) is the standard treatment for HER2+ breast cancer patients. Although there have been several insights into improved HER2 targeting and considerable efficacy shown by target agents, some patients have shown no response to treatment with others eventually progressing 12. These insights include inhibition of the HER family dimerization; which is an established mechanism of anti-HER2 therapy resistance by patients 13, delivering HER2-targeted chemotherapy where antibody-drug conjugate TDM1 (a combination of trastuzumab and emtansine) is used to deliver cytotoxic therapy directly to breast cancer cells 14, targeting HER2 / ER crosstalk which has been shown to promote tumor resistance in HER2+ / ER+ tumors 15 and the use of mutated HER2 as a target in HER2 non-amplified breast cancer in patients where somatic mutation in HER 2 gene is responsible for the activation of HER2 signaling pathway independent of dimerization of receptors 16. Ginkgo biloba nut has been used in traditional Chinese medicine for the management and treatment of varied medical conditions such as asthma and cough. It is also being used in the negation and treatment of brain, systemic circulatory disorders, and Alzheimer's disease 17. In addendum, pharmacological properties exhibited by Ginkgo biloba phytochemicalsinclude cell cycle regulatory, antioxidant, antiproliferative, anti-angiogenic and antiestrogenic activities 18.

Despite the great efficacy shown by drugs in the clinic against HER2 positive breast cancer, the reported resistance in patients with long-term trastuzumab treatment must be overcome and there is a need to identify and develop a novel therapeutic agent that can decrease the amount of HER2 in breast cancer cells which at the same time will possess a molecular mechanism of pharmacological activity that will subdue the resistance mechanisms employed against known agents.

## Methodology

### Ligand selection and preparation

The chemical structures of twenty-five phytochemicals were obtained from the database of PubChem compounds (https://pubchem.ncbi.nlm.nih.gov).The downloaded MOL SDF format of these ligands was converted to PDBQT file using PyRx tool to generate atomic coordinates and energy was minimized by using the optimization algorithm at force field set at mff (required) on PyRx.

### Accession and preparation of the target protein

The HER2 protein was prepared by recovering the threedimensional crystal structure of HER2 (PDB: 5o4g) in a complex with a cocrystallized ligand from RCSB PDB (Protein Data Base). (http://www.rcsb.org/pdb/home/home.do). Removing the bound complex molecule, non-essential water molecules and all heteroatoms using the Pymol toolkit then cleaned the protein. The co-crystallized ligand was extracted (not removed) from the active site to reveal the coordinate of the grid around the binding pocket when viewed on the pymol.

### Molecular docking using PyRx

After preparation of the receptor and ligands, molecular anchor (docking) analysis was done by PyRx, AutoDockVina option based on the notation functions. For our analysis, we used the exhaustive search anchoring function of PyRx, AutoDock Vina. After the minimization process, the grid resolution was centred at 76.6051 x 90.6294 x 55.7857 along x, y, and z-axes, respectively, for a grid size of 25x25x25 Å to defined the binding site. The cocrystallized binder (ligand), which serves as the default was first anchored on the linking (binding) site of HER2, and the resulting interaction was compared to that cianidanol in similar active sites using the same grid box dimension.

### Validation of docking results

The results obtained were validated during the blasting of the FASTA sequence of the HER2 crystalline structure (ID: 5o4g) obtained from the protein database of ChEMBL (Www.ebi .ac.uk/chembl/). The bioactivity generated by the database, with an activity of 64, an IC50 value of 1206 and the KI value of 178 was downloaded in txt format. Missing or lost data was removed only 30 of the 1206 drug-related compounds were recovered. The compiled compounds were split and converted to 2D (in sdf format) by the DataWarrior software (version 2) and converted to pdbqt format by the PyRx tool. The binders were anchored in the HER2 binding domain using the PyRx AutoDockVina logging function. A correlation coefficient was plotted between the coupling scores of 30 generated compounds and their corresponding PCHEMBL_VALUE values (determined experimentally). The graph of the correlation coefficient of Spearman Rank was plotted to obtain the correlation (R^2^) between the ChEMBL compounds and their corresponding results generated experimentally.

## Results and Discussion

**Figure 1:**
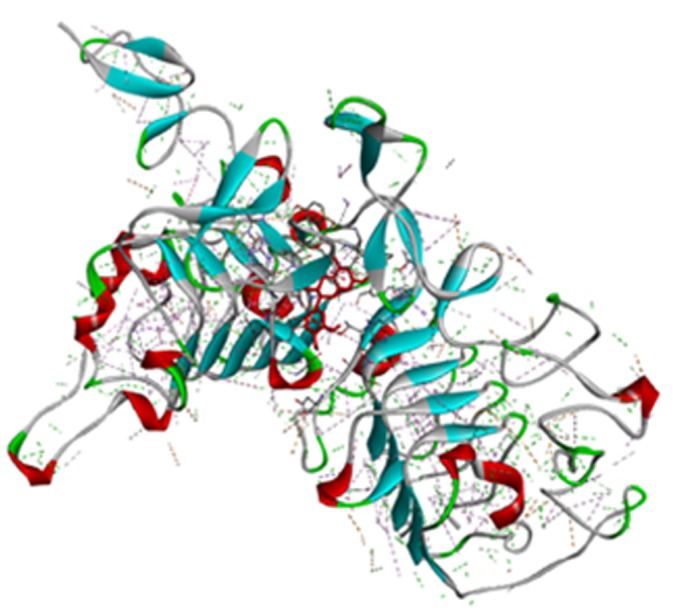
Structure of HER2+ (PDB ID: 5o4g) with cianidanol (red sticks). This image was generated using Discovery studio software.

Human Epidermal Growth Factor Receptor type 2 (HER2), belongs to the family of oncogenic proteins whose inhibition of amplification or over-expression has been shown to be cancer target (Figure 1). In the present study, twenty-five phytocompounds from Ginkgo biloba nut were docked into the binding pocket of HER2 (5o4g) for their HER2 (5o4g) inhibitory (antagonistic) properties. Cianidanol was discovered as the lead compound with the binding energy of -8.2 kcal/mol ( Table 1). The drug-likeness of cianidanol was assessed by subjecting it to the Lipinski's rule, Ghose's, Oprea's, Varma's and Verbier's rules. Cianidanol, the lead compound expressed significant 100%, 100%, 66.67%, 80%, 100% matches for Lipinski's rule, Ghose's, Oprea's, Varma's and Verbier's rules respectively, this describes its bioavailability and binding potential (Table 2).

**Figure 2:**
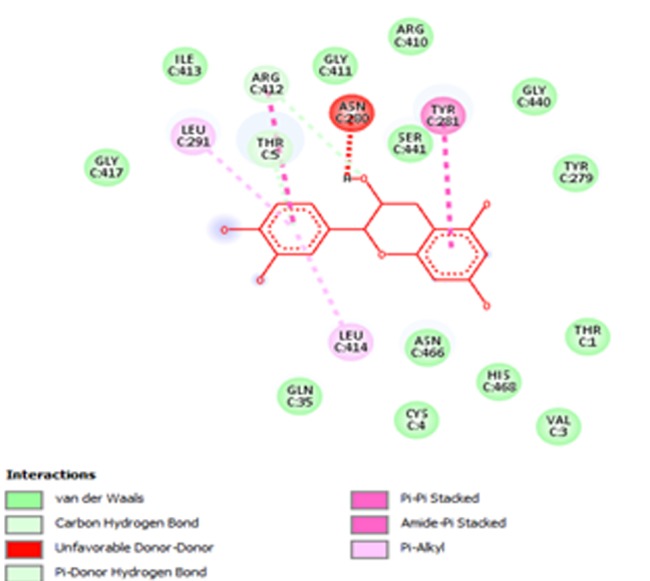
Interactions of cianidanol (red sticks) within the binding pocket of HER2+

Cianidanol, the lead compound has a binding energy of - 8.2kcal/mol, while the standard compound has a binding energy of -5.9kcal /mol (Table 1). The highest binding energy (- 8.2kcal/mol) attributed to cianidanol in this regard is believed tobe as a result of its chemical interactions at the receptor's active site (Table 3, Figure 2) which includes: Nineteen (19) Hydrogen bonds involving amino acids: T1, T5, V3, G6, Q35, C4, N280, R410, Y281, P278, R412, N280, N466, G417, G411, L414, S441, R410, H468, L291. Thirteen (13) hydrophobic interactions involving amino acids: L291, L414, I413, Y281, R412, R410, P278, H468, C4. While that of the co-crystallized ligand (PDB Ligand ID: NAG), which serves as standard, presents the following chemical interactions at the binding pocket (Table 4). Twelve Hydrogen bonds involving amino acids: T1, V3, N280, N466, R410, R412, T281, P278, S441, G411, H468, L291. Eight Hydrophobic interactions involving amino acids: L291, Y281, Y278, F269, R410, R412, R465, R278, H468 and C4.

Though the compound with PUBCHEM ID: 5271805 (Ginkgetin) as the highest binding energy (-9.5kcal/mol), it failed the ADME evaluation test. Cianidanol, the next compound with the highest binding energy (-8.2 kcal/mol), is thought to be the result of the large number of hydrophobic interactions (thirteen hydrophobic interactions) compared to the seven in the co-crystallized ligand interactions with the binding pocket. Hydrophobic interactions can increase the binding affinity between target-drug interfaces 16.

**Figure 3:**
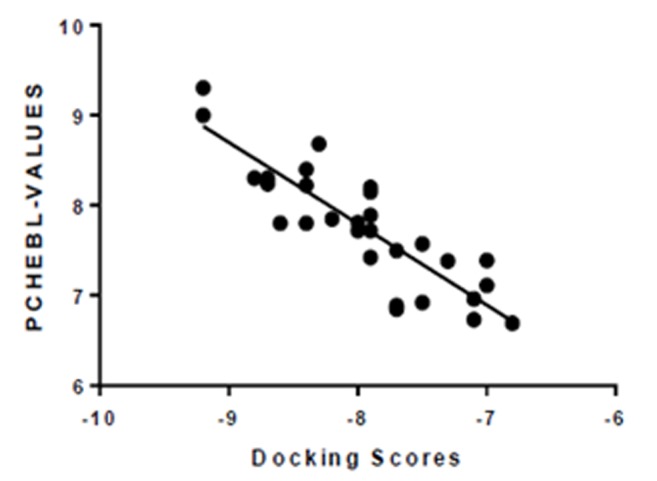
Correlation coefficient graph of docking scores of various antagonists of the HER2 and their corresponding experimental IC50 (Pchembl_values) values obtained from CHEBL database (www.ebi.ac.uk/chembl/)

The reliability of our docking scores was validated using the online ChEMBL database, the Fasta sequence of the HER2 crystalline structure (ID: 5o4g) obtained was BLAST on www.ebi.ac.uk/chembl/. The binding site of HER was docked with the compounds obtained from the search, and a correlation coefficient graph was generated by plotting the CHEMBL's Pchem values (experimentally determined IC50) and the docking scores of the compounds obtained from the search. From the plot a strong correlation coefficient of (R^2^ = 0.75) was obtained (Figure 3), this gives certitude to the verity and reliability of the computational experiment and that PyRx Auto DockVina algorithm is dependable (Table 1).

## Conclusion

Docking studies and ADMET evaluation of cianidanol showed that this ligand is drug-gable and plays critical role in the inhibition of HER2. It could be deduced that cianidanol could service as a potential antagonistic agent against HER2+, which is overexpressed in aggressive female breast cancer.

## Figures and Tables

**Table 1:** Interaction table showing the various chemical interactions of cianidanol within the binding pocket (Viewed on Discovery studio Visualizer)

S/N	PubChem CID of Ligands	E-values	Binding Affinity	Rmsd per ub	Rmsd per lb
1Standard (NAG)	E=443.47	-5.9	0	0
272	E=3.45	-5.4	0	0
31064	E=1595.37	-5.8	0	0
45991	E=1741.84	-7.4	0	0
58468	E=100.10	-5.5	0	0
69064	E=1486.05	-8.2	0	0
765084	E=1478.50	-7.5	0	0
872277	E=1478.50	-7.6	0	0
991457	E=10414.30	-8	0	0
1092138	E=4665.85	-7.4	0	0
11107876	E=1090.60	-7.9	0	0
12108065	E=177.37	-8	0	0
13163776	E=1137.50	-8	0	0
14182232	E=1486.05	-7.4	0	0
15296119	E=3680.28	-8.1	0	0
16443023	E=122.42	-6.8	0	0
175165850	E=2027.66	-5.6	0	0
185271805	E=176.23	-9.5	0	0
19637542	E=-21.04	-5.8	0	0
20638014	E=1008.71	-6.3	0	0
215280442	E=53.87	-8	0	0
225280443	E=35.86	-8.1	0	0
235280445	E=42.52	-8.1	0	0
245280863	E=61.21	-8.1	0	0
255281654	E=84.09	-7.4	0	0

**Table 2:** Lipinski's, Ghose's, Opera's, Varma's and Verber's drug-like properties of cianidanol: The rules describes drug pharmacokinetics in the human body which also including their absorption, distribution, metabolism, and excretion ('ADME') using an online server (http://admet.scbdd.com). MW= Molecular weight, Hacc= Hydrogen acceptor, Hdon= Hydrogen donor, MR= Molar Refractivity, natoms=number of atoms, nRotbound= Number of ratable bound, TPSA= Topological surface area. N= Number.

Lipinski's Rule					Ghose's Rule	Opera's Rule	Varma's Rule	Verber's Rule
Lipinski�s Rule					Ghose's Rule	Opera's Rule	Varma's Rule	Verber's Rule
IUPAC Name	SMILES	PubChem CID						
Cianidanol	C1C2C(COC2C3=CC4=C(C=C3)OCO4)C(O1)C5=CC6=C(C=C5)OCO6	9064					Matches (percent)	
MW	HBD	HBA	LogP	Matches				
290.271	5	6	1.546	100 percent	100 percent	66.67 percent	80 percent	100 percent
Lipinski's Rule	Ghose' s Rule	Opera�s Rule	Varma' s Rule	Verber's Rule				
Molecular properties	Molecular properties	Molecular properties	Molecular properties	Molecular properties	Ghose	Opera	Varma	Verber�s
MV ≤ 500	-5.6 < McLog P < -0.4 Mean = 2.52	nrings=3	MW≤ 500	nRotbond=12	1.546	3	290.271	1
LogP ≤ 5	160< MW < 480 Mean=357	nrigidbond=18	TPSA ≤ 125	TPSA ≤ 140	290.271	22	110.38	110.38
Hacc ≤ 10	40 < MR < 130 Mean=97	nRotbond=6	-5 < LogD < -2	Hacc and Hdon = 12	72.623	1	0.115	11
Hdon ≤ 5	20 < natoms < 70 Mean = 48		Hacc and Hdon=9		35		11	
			nRotbond=12				1	

**Table 3:** Interaction table showing the various chemical interactions of Cianidanol within the binding pocket

Name	Category	Types
C:T1:HG1 - C:V3:O	Hydrogen Bond	Conventional Hydrogen Bond
C:G6:HN - C:Q35:O	Hydrogen Bond	Conventional Hydrogen Bond
C:Q35:HN - C:C4:O	Hydrogen Bond	Conventional Hydrogen Bond
C:N280:HD21 - C:R410:O	Hydrogen Bond	Conventional Hydrogen Bond
C:T281:HN - C:P278:O	Hydrogen Bond	Conventional Hydrogen Bond
C:R412:HE - C:N280:O	Hydrogen Bond	Conventional Hydrogen Bond
C:R412:HE - C:N280:OD1	Hydrogen Bond	Conventional Hydrogen Bond
C:G417:HN - C:L414:O	Hydrogen Bond	Conventional Hydrogen Bond
C:S441:HN - C:R410:O	Hydrogen Bond	Conventional Hydrogen Bond
C:S441:HG - C:G411:O	Hydrogen Bond	Conventional Hydrogen Bond
C:H468:HN - C:N466:OD1	Hydrogen Bond	Conventional Hydrogen Bond
C:H468:HE2 - C:T1:O	Hydrogen Bond	Conventional Hydrogen Bond
C:H468:HE2 - C:T1:OG1	Hydrogen Bond	Conventional Hydrogen Bond
C:R410:CD - C:N280:OD1	Hydrogen Bond	Carbon Hydrogen Bond
C:R412:CA - N:UNK1:O	Hydrogen Bond	Carbon Hydrogen Bond
C:R412:CD - C:L291:O	Hydrogen Bond	Carbon Hydrogen Bond
C:T5:HG1 - N:UNK1	Hydrogen Bond	Pi-Donor Hydrogen Bond
C:L291:CD2 - C:T281	Hydrophobic	Pi-Sigma
C:T281 - N:UNK1	Hydrophobic	Pi-Pi Stacked
C:R412:C,O;I413:N - N:UNK1	Hydrophobic	Amide-Pi Stacked
C:R410 - C:R412	Hydrophobic	Alkyl
C:R412 - C:L291	Hydrophobic	Alkyl
C:T281 - C:P278	Hydrophobic	Pi-Alkyl
C:H468 - C:C4	Hydrophobic	Pi-Alkyl
N:UNK1 - C:L291	Hydrophobic	Pi-Alkyl
N:UNK1 - C:L414	Hydrophobic	Pi-Alkyl
C:R412:CA - N:UNK1:O	Hydrogen Bond	Carbon Hydrogen Bond
C:T5:HG1 - N:UNK1	Hydrogen Bond	Pi-Donor Hydrogen Bond
C:T281 - N:UNK1	Hydrophobic	Pi-Pi Stacked
C:R412:C,O;I413:N - N:UNK1	Hydrophobic	Amide-Pi Stacked
N:UNK1 - C:L291	Hydrophobic	Pi-Alkyl
N:UNK1 - C:L414	Hydrophobic	Pi-Alkyl

**Table 4:** Interaction table showing the chemical interaction of the co-crystalized Ligand within the binding pocket

Name	Category	Types
C:T1:HG1 - C:V3:O	Hydrogen Bond	Conventional Hydrogen Bond
C:N280:HD21 - C:R410:O	Hydrogen Bond	Conventional Hydrogen Bond
C:T281:HN - C:P278:O	Hydrogen Bond	Conventional Hydrogen Bond
C:R412:HE - C:N280:O	Hydrogen Bond	Conventional Hydrogen Bond
C:R412:HE - C:N280:OD1	Hydrogen Bond	Conventional Hydrogen Bond
C:S441:HN - C:R410:O	Hydrogen Bond	Conventional Hydrogen Bond
C:S441:HG - C:G411:O	Hydrogen Bond	Conventional Hydrogen Bond
C:H468:HN - C:N466:OD1	Hydrogen Bond	Conventional Hydrogen Bond
C:H468:HE2 - C:T1:O	Hydrogen Bond	Conventional Hydrogen Bond
C:H468:HE2 - C:T1:OG1	Hydrogen Bond	Conventional Hydrogen Bond
C:R410:CD - C:N280:OD1	Hydrogen Bond	Carbon Hydrogen Bond
C:R412:CD - C:L291:O	Hydrogen Bond	Carbon Hydrogen Bond
C:L291:CD2 - C:T281	Hydrophobic	Pi-Sigma
C:F269 - C:T281	Hydrophobic	Pi-Pi T-shaped
C:R410 - C:R412	Hydrophobic	Alkyl
C:R412 - C:L291	Hydrophobic	Alkyl
C:F269 - C:P278	Hydrophobic	Pi-Alkyl
C:T279 - C:R465	Hydrophobic	Pi-Alkyl
C:T281 - C:P278	Hydrophobic	Pi-Alkyl
C:H468 - C:C4	Hydrophobic	Pi-Alkyl
